# Effects of Coconut Exocarp Flavonoid and EDTA-2Na on Aldehyde Generation During Pan-Frying Processing of Squid (*Dsidicus gigas*)

**DOI:** 10.3390/foods14111925

**Published:** 2025-05-28

**Authors:** Xinwen Wang, Hongping Lin, Mantong Zhao, Yuehan Lu, Guanghua Xia, Zhongyuan Liu

**Affiliations:** 1 School of Food Science and Engineering, Hainan University, Haikou 570228, China; wxwbfq@163.com (X.W.); linhongping@163.com (H.L.); mantongzhao@126.com (M.Z.); luyuehan@hainanu.edu.cn (Y.L.); xiaguanghua2011@126.com (G.X.); 2 Hainan Provincial Engineering Research Centre of Aquatic Resources Efficient Utilization in the South China Sea, Haikou Key Laboratory of Deep Processing of Marine Food, Haikou 570228, China

**Keywords:** squid, pan-frying, lipid oxidation, aldehydes, flavonoids

## Abstract

Squid is rich in polyunsaturated fatty acids (PUFAs), especially docosahexaenoic acid (DHA) and eicosapentaenoic acid (EPA), which exert various human health benefits. Pan-fried squid is a popular processed product beloved by consumers. However, the PUFAs of squid can be severely oxidized during thermal processing, which will result in the reduction in nutritional value and generation of harmful compounds like aldehydes. In this study, flavonoids extracted from coconut exocarp (CEF) and the metal ion chelating agent disodium ethylenediaminetetraacetate (EDTA-2Na) were used to inhibit lipid oxidation during the frying of squid, with the lipid oxidation level, the changes in fatty acid composition, and aldehyde concentrations being examined by gas chromatography mass spectrometry and high-performance liquid chromatography mass spectrometry. Results indicated that during pan-frying, the peroxide value, thiobarbituric acid value, and total oxidation value increased significantly, while the contents of EPA and DHA decreased significantly, and the concentrations of most aldehydes increased in a time- and temperature-dependent pattern. Both CEF and EDTA-2Na treatments inhibited these changes; comparatively, the CEF treatment was significantly better than that of EDTA-2Na. For instance, the CEF treatment inhibited the generation of HHE by 31.90%, 33.24%, and 19.73%, respectively, after pan-frying of squid at 180 °C for 6, 8, and 10 min, while the corresponding values for HNE were 22.65%, 18.96%, and 17.28% respectively. These results suggested that CEF can improve the oxidative stability of squid lipids during pan-frying and reduce the generation and accumulation of aldehydes and improve the security of processed squid products.

## 1. Introduction

For a long time, marine organisms have received extensive attention because they are rich in polyunsaturated fatty acids (PUFAs), especially the omega-3 PUFA (n-3 PUFAs). Since the 20th century, ever-increasing studies have found that n-3 PUFA, represented by eicosapentaenoic acid (EPA) and docosahexaenoic acid (DHA), possess a variety of physiological benefits, such as improving mitochondrial function [1], improving memory [2], promoting brain development [3], reducing the risk of cardiovascular disease [4], diabetes [5], cancer [6], and depression [7]. In recent years, with the development of the aging population and the increasing burden of chronic diseases, the nutritional value of EPA and DHA has been further emphasized, and the development and utilization of marine lipids has become a hot research topic.

While marine lipids have the above benefits, they also face serious challenges. Under thermal processing (frying, steaming, baking) of aquatic products, the PUFAs are vulnerable to oxidation by the high temperatures and prolonged processing time. The presence of transition metal ions can also accelerate the PUFA oxidation through free radical chain reactions to generate unstable hydroperoxides, which are prone to further degradation into small molecules such as aldehydes [8]. This process not only results in significant loss of nutritional value of the food but also poses potential risks to human health. It has been demonstrated that certain reactive aldehydes readily react with biomacromolecules, such as proteins, amino acids, and nucleic acids [9]. This reaction compromises the functional integrity of biomacromolecules, generates cytotoxicity, and induces apoptosis [10]. In particular, α, β-unsaturated aldehydes have been implicated in the progression of various diseases, such as atherosclerosis [11], cancer [12], neurodegenerative disorders [13], and multiple sclerosis [14]. Therefore, the inhibition of lipid oxidation in aquatic products during processing has become the focus of research in recent years.

Squid is a strategic fishery resource with both nutritional and economic value. Squid is rich in PUFA, especially EPA and DHA, which can reach more than 30% of the total lipid and is a high-quality n-3 PUFA dietary supplement source. Pan-frying is a common processing method for squid that endows them with attractive color and flavor. Fried squid products are widely popular worldwide, especially in coastal areas. However, during the pan-frying, squid lipids are susceptible to oxidation and generate some harmful aldehydes, which seriously affects the nutritional value and safety. In recent years, reactive aldehydes derived from PUFA oxidation of aquatic products under thermal processing have been extensively studied. Massive aldehyde contents from oxidative degradation of lipids in oysters [15], salmonids [16], golden pompano and clam [17,18] upon different thermal processing methods (roasting, boiling, frying, and air frying) have been observed. However, little information was available about the lipid oxidation of squid during thermal processing.

Natural antioxidants, like polyphenols, are extensively utilized to inhibit lipid oxidation during thermal processing due to their ability to scavenge free radicals, inhibit pro-oxidant enzyme activities, and chelate metal ions. For example, polyphenols in apple pomace could inhibit lipid oxidation in French fries [19]. Black chokeberry fruit polyphenols were observed to reduce the PUFA oxidation and inhibit aldehyde production [20]. Rosemary can inhibit the formation of 4-hydroxy-nonenal (HNE) from hydroperoxide decomposition under salmon frying processing [21]. In our previous study, flavonoids extracted from coconut exocarp (CEF) were observed to hinder the lipid oxidation of golden pompano fillets during cold storage and extend the shelf-life [22]. Moreover, some polyphenol compounds like quercetin could exert the ability to capture the aldehydes by addition reaction [23]. These results confirmed the potential of natural antioxidants in inhibiting lipid oxidation during thermal processing.

Therefore, in this study, flavonoids extracted from coconut exocarp (CEF) and the metal ion chelating agent disodium ethylenediaminetetraacetate (EDTA-2Na) were used to inhibit lipid oxidation during the pan-frying of squid (*Dosidicus gigas*). The lipid oxidation level, the changes in fatty acid composition, and aldehyde concentrations were investigated by gas chromatography mass spectrometry (GC-MS) and high-performance liquid chromatography mass spectrometry (HPLC-MS/MS), aiming to seek effective measures for inhibition of harmful aldehyde generation and enhance squid consumption safety for consumers.

## 2. Materials and Methods

### 2.1. Materials

Three kilogram of fresh squid (*Dosidicus gigas*), fresh coconut (*Cocos nucifera*), and corn oil were purchased from a local market in Haikou, Hainan, China. Fresh mixed fatty acid methyl ester standards within C4-C24 carbons were obtained from Experimental Technology Co., Ltd. (Shanghai, China). Acrolein, propanal, butanal, and other aldehyde standards were obtained from Bedoukian Research, Inc. (Danbury, CT, USA). Hexane, dichloromethane, methanol, trichloromethane, 2,4-dinitrophenylhydrazine (DNPH), and other reagents were obtained from Xilong Science Co., Ltd. (Shantou, China).

### 2.2. Sample Preparation

The head and viscera of the squid were removed, and the squid muscle was retained and cleaned for pan-frying. The CEF was extracted based on the method in our previous research [24]. The squid meat was divided into four groups: fresh, control, EDTA-2Na group, and CEF group. Except for the fresh sample, the squid meat was treated with deionized water, 0.02% (*m*/*v*) of EDTA-2Na solution, and 0.2% (*m*/*v*) of CEF solution marinade, respectively, for 2 h at room temperature. Afterward, each group of samples was randomly divided into six portions and pan-fried with corn oil at 160 °C and 180 °C for 6 min, 8 min, and 10 min. At the end of the frying process, the surface oil of the squid was drained as much as possible. Finally, the obtained squid samples were freeze-dried with a vacuum freeze dryer (Beijing Songyuan Huaxing Technology Development Co., Ltd., Beijing, China) and pulverized using a multifunctional pulverizer (Deqing Baijie Electric Co., Ltd., Hangzhou, China) to obtain the powder.

### 2.3. Lipid Extraction from Squid

Lipids were extracted from lyophilized powder of squid based on the Bligh–Dyer method [25]. The extracted squid lipids were weighed and recorded and subsequently stored at −80 °C for further analysis.

### 2.4. Determination of Peroxide Value (PV)

Method Cd 8b-90 of the American Oil Chemists’ Society was referenced and slightly modified for the detection of PV changes in squid lipids during frying [26]. Briefly, 200 mg of squid lipids was added to 50 mL of a mixture of glacial acetic acid/isooctane (*v*/*v* = 3:2) and titrated to the end point with 0.005 M Na_2_S_2_O_3_. PV measurements of squid lipids expressed as meq/kg lipid.

### 2.5. Determination of Thiobarbituric Acid-Reactive Substances (TBARS)

TBARS of squid lipids extracted by Bligh–Dyer method was determined by the method reported by Hu et al. [27] with minor modifications. Briefly, absorbance values were determined using 500 mg of squid lipids, and the results were calculated by taking the standard curve constructed from a solution of malondialdehyde (MDA) precursor (1,1,3,3-tetramethoxypropane). TBARS measurements of squid lipids expressed as mg MDA eq/kg lipid.

### 2.6. Determination of Total Oxidation Value (TOTOX)

TOTOX was calculated according to the method proposed by Shahidi and Wanasundara in 1995 [28]. TOTOX combines the primary oxidation index, PV, and the secondary oxidation index, TBARS, which is often used to reflect the comprehensive status of lipid oxidation.

### 2.7. Fatty Acid Composition Analysis

The fatty acid composition of squid was determined using the method reported by Yan et al. [29] with slight modification. The procedure was as follows: 50 mg of squid lyophilized powder was added to 1 mL of dichloromethane/methanol mixture (*v*/*v* = 1:1), centrifuged for 15 min, and then 500 μL of supernatant was taken in a test tube and blown dry under N_2_. Then, 0.5 mL of 0.5 M sodium hydroxide methanol solution was added into the test tube for reaction under 60 °C water baths for 1 h. Finally, 0.5 mL of hexane was added and centrifuged for 15 min to take 100 μL of the upper layer of the solution for filtering through a 0.22 μm organic-phase membrane before injection into GC-MS for analysis. The fatty acid composition was calculated from the standard curve of the fatty acid methyl ester specimen and expressed as relative content (%).

The squid samples were analyzed using an Agilent 8890-7000D GC/MSD instrument (Santa Clara, CA, USA). The chromatographic column was an Agilent DB-Fast FAME capillary column (20 m × 0.18 mm × 0.2 μm). The injection volume was 1.0 μL, and the split ratio was 50:1. The initial temperature was 80 °C for 0.5 min, and then the temperature was increased to 175 °C (70 °C/min), and then to 230 °C (8 °C/min), which was maintained for 1 min, and then maintained at 80 °C for 2 min. The carrier gas used was high-purity helium (>99.99%). Ion scanning mode was selected for MS analysis, with electron bombardment of the ion source. The electron energy was 70 eV, the temperature of ion source was 230 °C, the temperature of quadrupole was 150 °C, and the temperature of transmission line was 240 °C.

### 2.8. Aldehyde Content Analysis

The aldehyde contents in squid lipids were determined using the method reported by Zhao et al. [15] with slight modification. The procedure was as follows: 200 mg of squid lipid was added to 2 mL of 10 mg/mL DNPH solution (dissolved in a mixture of acetonitrile/formic acid = 99:1). After vortexing for 2 min and mixing thoroughly, the reaction was carried out in a dark room at 40 °C for 2 h under the water bath condition. At the end of the reaction, the solution was added to 5 mL with acetonitrile and injected into the sample vial after passing through a 0.22 μm organic phase membrane. The aldehyde contents of squid were calculated from the standard curves and expressed as μg/g lipid.

The aldehyde contents in the squid lipids were determined using a Shimadzu LC-40AVP system (Tokyo, Japan) equipped with a Phenomenex Kinetex C18 column (150 mm × 2.1 mm, 1.7 μm, Torrance, CA, USA), based on our previous study. The mobile phase was A: 0.1% formic acid aqueous solution; B: 0.1% formic acid in acetonitrile; the flow rate was 0.3 mL/min with an injection volume of 5 μL; the gradient elution program was as follows: 0–2 min, 40–46% B; 2–20 min, 46–83% B; 20–22 min, 83–90% B; 22–24 min, 90–40% B; 24–25 min, 40% B.

MS analysis was performed using an QTRAP6500+ triple quadrupole mass spectrometer (AB Sciex, Foster City, CA, USA) in negative ion mode with parameters as follows: ion source temperature: 425 °C, ionspray voltage: −4500 V, ion source gas 1: 45 psi, ion source gas 2: 40 psi, declustering potential: −100 V, entrance potential: −12 V.

### 2.9. Statistical Analysis

All the experiments were conducted at least in triplicate, and all data were expressed as mean ± standard deviation (SD), analyzed using the one-way ANOVA test by SPSS Statistics 27 software, and image-related data were plotted using Origin 2024. Differences were evaluated by one-way analysis of variance (Student–Newman–Keuls post hoc test) considered significant at *p* < 0.05.

## 3. Results and Discussion

### 3.1. Changes in Oxidation Indices

**Figure 1:**
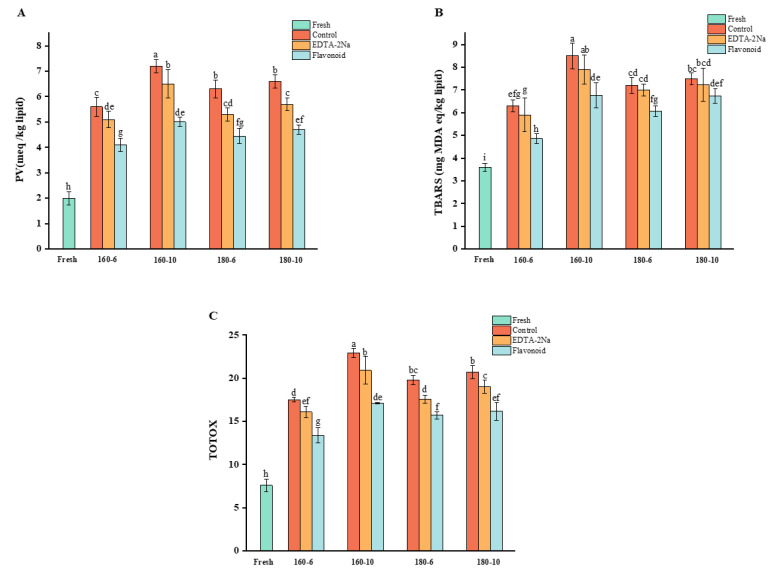
Changes in PV (**A**), TBARS (**B**), and TOTOX value (**C**) of squid lipids during frying. Fresh, 160-6, 160-10, 180-6, and 180-10 represent samples of fresh squid, fried at 160 °C for 6 min, 160 °C for 10 min, 180 °C for 6 min, and 180 °C for 10 min, respectively. Different letters (a–i) in each figure (**A**–**C**) represent statistically significant differences (*p* < 0.05) between different squid lipid samples.

Changes in oxidation indexes during frying of squid are shown in Figure 1, including PV, TBARS, and TOTOX.

#### 3.1.1. Change in Peroxide Value

Hydroperoxides are produced during the primary stages of lipid oxidation; therefore, PV is often used to reflect the extent of lipid oxidation [8]. As shown in Figure 1A, the PV of fresh squid was 2.07 meq/kg lipid. All fried squid samples showed significantly higher PV than fresh squid, and the highest PV, 7.26 meq/kg lipid, was observed in the control group when fried at 160 °C for 10 min. During frying at 160 °C, the PV showed obvious time dependence, and the PV increased significantly with time. These results suggested that the pan-frying process caused severe oxidation of squid lipids, hydroperoxide formation, and accumulation. Consistent with our result, Multari et al. [30] found that the PV of soybean oil increased significantly with the frying time; Bejaou et al. [31] found that the PV of clam lipids increased significantly during pan-frying. However, during frying at 180 °C, the PV increased with time, but the increase was not significant, and the PV at 180 °C for 10 min was significantly lower than the PV at 160 °C for 10 min. This was due to the rapid decomposition of unstable hydroperoxides generated by lipid oxidation of squid into secondary oxidation products during prolonged frying at high temperatures. This was consistent with the findings of previous studies: Manzoor et al. [32] reported that there is a tendency for the PV of chicken and fish lipids to increase and then decrease during frying. Andreo et al. [33] found that the PV of pork and beef lipids changed sinusoidally during heating.

#### 3.1.2. Change in Thiobarbituric Acid-Reactive Substances Value

Hydroperoxides are extremely unstable and are further decomposed to produce aldehydes, ketones, and acids, of which MDA is used to express TBARS [8]. As shown in Figure 1B, the TBARS of fresh squid was 3.67 mg MDA eq/kg lipid. All fried squid samples showed significantly higher TBARS than fresh squid, and the highest TBARS, 8.47 mg MDA eq/kg lipid, was observed in the control group when fried at 160 °C for 10 min. The changes in TBARS of squid lipids exhibited a similar trend to PV. Under identical temperature conditions, the TBARS values of squid lipids increased over time. Specifically, the TBARS values for frying at 10 min were consistently higher than those for frying at 6 min. Under identical time conditions, the TBARS values of squid lipids were significantly higher when fried at 180 °C for 6 min compared to frying at 160 °C for the same duration. However, the TBARS values of squid lipids were significantly lower when fried at 180 °C for 10 min compared to frying at 160 °C for 10 min. This phenomenon can be traced to two causes: On the one hand, an increase in frying temperature exacerbates lipid oxidation and generates secondary oxidation products, leading to an increase in TBARS values of squid lipids [34]. On the other hand, prolonged frying at high temperatures led to the volatilization of low molecular aldehydes; the reaction of aldehydes with proteins, amino acids, and nucleic acids could also result in the decrease in TBARS values of squid lipids [35]. The reliability of this result was confirmed by the results of previous studies. Negara et al. [36] found that the frying process significantly increased the TBARS of chub mackerel (*Scomber japonicus*). Katragadda et al. [37] reported that the content of volatile carbonyl compounds increases with temperature and time, but instead of a sustained increase, there was an inflection point due to increased lipid oxidation and reactions occurring in the oxidation products.

#### 3.1.3. Change in Total Oxidation Value

TOTOX combines the primary oxidation index PV and the secondary oxidation index TBARS for a comprehensive assessment of the degree of lipid oxidation [8]. As shown in Figure 1C, the TOTOX of fresh squid was 7.59. All fried squid samples showed significantly higher TOTOX than fresh squid, and the highest TOTOX value of 22.93 was observed in the control group when fried at 160 °C for 10 min. The changes in TOTOX value of squid lipids exhibited a similar trend with PV and TBARS. However, the increment of TOTOX of squid lipids during the frying process at 180 °C was significantly lower than that at 160 °C. These results confirmed the occurrence of lipid oxidation in squid during pan-frying process.

The use of antioxidants is an important means of inhibiting lipid oxidation during thermal processing. As shown in Figure 1, both CEF and EDTA-2Na treatments inhibited the rise of various lipid oxidation indices in squid, and the effect of CEF treatment was significantly better than that of EDTA-2Na treatment. Specifically, CEF treatment showed significant inhibition effects at all temperatures and time conditions, while EDTA-2Na treatment showed an insignificant inhibition effect under some conditions. For example, there was no significant difference in TBARS between EDTA-2Na-treated and control group samples at 180 °C for 6 min. Furthermore, the inhibitory effect of EDTA-2Na treatment on lipid oxidation was significantly lower than that of CEF treatment under all time and temperature conditions (*p* < 0.05). These results indicated that CEF, as a natural antioxidant, possesses excellent antioxidant effects and can significantly inhibit lipid oxidation during the frying process of squid.

### 3.2. Changes in Fatty Acid Composition

As shown in Table 1 and Table 2, 21 fatty acids, including 4 monounsaturated fatty acids, 7 PUFAs, and 10 saturated fatty acids, were detected in fresh squid lipids. Among them, C16:0 (palmitic acid), C18:0 (stearic acid), C20:4 n-6 (arachidonic acid), C20:5 n-3 (EPA), and C22:6 n-3 (DHA) were abundant, accounting for 33.28%, 10.63%, 7.70%, 8.93%, and 28.37% of the total fatty acid, respectively. The PUFA content in fresh squid accounted for 45.78% of the total fatty acids, and the sum of EPA and DHA accounted for 37.30% of the total fatty acids, which was consistent with previous studies [38,39]. Fatty acids including C16:0, C18:0, C20:1 n-9, C20:4 n-6, EPA, and DHA were the major fatty acids in fresh squid, with the PUFA content accounting for about 50% of the total fatty acid content and the sum of the EPA and DHA content accounting for 30–40% of the total fatty acid content [38,39]. These reports proved the reliability of squid as a good source of n-3 PUFAs from daily diet supplements.

The changes in fatty acid composition are a key indicator to determine the lipid oxidation of squid during frying, which is significant for evaluating the nutritional value of squid. As shown in Table 1 and Table 2, the relative contents of C18:1 n-9 (oleic acid) and C18:2 n-6 (linoleic acid) in the fatty acids of squid increased significantly during frying, especially C18:2 n-6, which increased from 0.25% to 45–50%. However, the relative contents of palmitic acid, stearic acid, arachidonic acid, EPA, and DHA decreased significantly, especially DHA, which decreased by 75.26% and 83.08% at 160 °C for 6 min and 10 min of frying, respectively, and decreased by 84.21% and 80.93% at 180 °C for 6 min and 10 min of frying, respectively. In some previous studies, Li et al. [40] reported that the relative content of linoleic acid increased significantly, and the relative content of palmitic acid, stearic acid, arachidonic acid, EPA, and DHA decreased significantly in grass carp (*Ctenopharyngodon idellus*) during the frying process. By examining the fatty acid composition of tilapia (*Oreochromis niloticus*), Mekonnen et al. [41] found that frying treatment significantly reduced the relative contents of stearic acid, arachidonic acid, EPA, and DHA, especially DHA, which decreased from 7.67% to 0.87%. In addition, during the frying of anchovy (*Engraulis encrasicholus*) and sardine (*Rastrineobola argentea*), the significant decreases in relative EPA and DHA content and significant increases in relative linoleic acid content were also observed [42,43]. This was consistent with the expected results, since the number of double bonds affects fatty acid breakdown [44]. Therefore, the PUFAs of squid, especially EPA and DHA, are prone to being oxidized during the frying process. However, as shown in Table 1 and Table 2, the relative content of PUFAs in the squid samples did not decrease during the frying process but rather increased. This was due to the lipid exchange between squid and corn oil during frying, which significantly increased the relative content of linoleic acid, leading to an increase in the relative content of PUFA. This finding was validated in previous studies. Liu et al. [17] reported that lipid exchange occurs between clams and frying oil during frying and that major fatty acids from frying oil are enriched in fried clams (*Ruditapes philippinarum*). Naseri et al. [45] found that silver carp lipids were exchanged with the frying oil during frying, and the exchange was different for frying oils with distinct fatty acid compositions. In summary, frying treatment causes serious oxidative loss of squid PUFAs, especially EPA and DHA, which seriously affect the nutritional value of squid.

**Figure 2:**
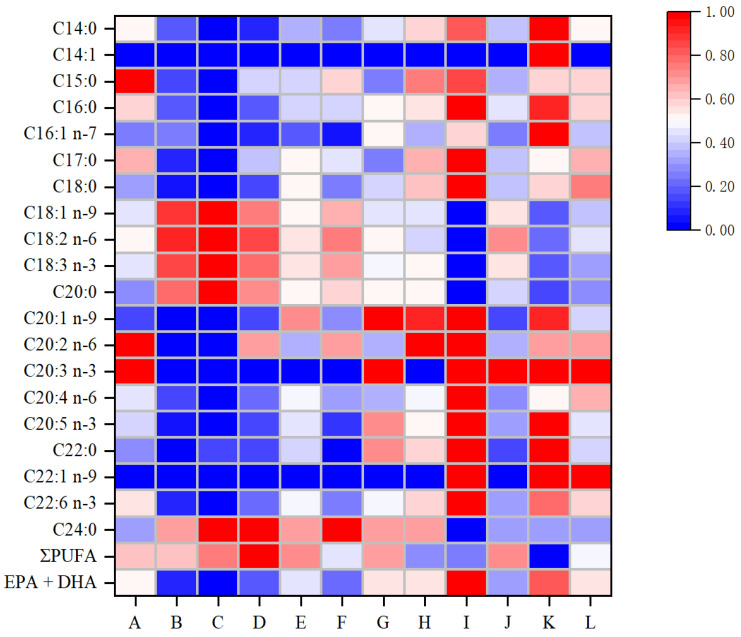
Heat map of changes in fatty acid composition in squid lipids. A–D represent the squid samples fried at 160 °C for 6 min and 10 min and at 180 °C for 6 min and 10 min, respectively. E–H represent the squid samples fried under the same conditions after treatment with EDTA-2Na. I–L represent the squid samples fried under the same conditions after treatment with CEF.

Consistent with the results of the squid oxidation indices, both CEF and EDTA-2Na treatments were able to inhibit the reduction in squid EPA and DHA contents during frying, and the protection effect of CEF on the loss of EPA and DHA was significantly better than that of EDTA-2Na. For example, as shown in Table 1, the relative EPA and DHA contents of CEF-treated squid were 1.39, 1.23 folds and 1.28, 1.24 folds higher than the control group at 160 °C frying treatment for 6 min and 10 min, respectively, and there was no significant difference in the relative EPA and DHA contents of EDTA-2Na-treated squid, although they increased. As shown in Table 2, the relative contents of EPA and DHA of CEF-treated squid were 1.93, 1.27 folds and 1.77, 1.31 folds higher than the control group, and the relative contents of EPA and DHA of EDTA-2Na-treated squid were 1.67, 1.33 folds and 1.50, 1.30 folds higher than the control group, respectively, at 180 °C frying treatments for 6 min and 10 min. This difference can be visualized in Figure 2. These results indicated that although EDTA-2Na treatment was able to inhibit the fatty acid oxidation rate in squid lipids through metal ion chelation, this inhibitory effect tended to be more significant when lipid oxidation was more severe (high temperature and long time). In contrast, CEF treatment showed significant inhibition of EPA and DHA oxidation in squid lipids under both conditions. Several previous studies have also reported the excellent role of natural polyphenols in inhibiting fatty acid oxidation. Wang et al. [46] reported that polyphenols were able to maintain the fatty acid composition of frying oils and inhibit the oxidation of fatty acids during frying processing. Li et al. [47] found that rosemary polyphenol extract could inhibit the oxidation of PUFAs in fried soybean oil. Hu et al. [27] found that polyphenols from bamboo leaves significantly inhibited fatty acid oxidation in the scallop frying process. Manzoor et al. [48] found that apple pomace polyphenol extracts significantly reduced the rate of PUFA oxidation in soybean oil during deep frying of French fries. Taken together, these results indicated that CEF could enhance the oxidative stability of squid lipids and significantly inhibit the oxidative loss of squid PUFAs, especially EPA and DHA, during frying. This is of great significance for maintaining the nutritional value of squid.

### 3.3. Changes in Aldehyde Contents

As shown in Table 3 and Table 4, 12 target aldehydes were detected in the lipids of pan-fried squid. Preliminary observations revealed that the concentration of most aldehydes in squid lipids increased with frying time and temperature. Propanal, butanal, pentanal, 4-hydroxy-hexenal (HHE), and HNE were the main aldehydes in the lipids of fried squid, with concentrations higher than 15 μg/g. This was similar to the findings of Hu et al. [27], who found high concentrations of propanal, butanal, and pentanal during the frying process of scallops. The concentrations of other aldehydes were relatively low, especially the *trans*-2-hexenal, which was difficult to detect under some conditions. *Trans*-2-hexenal is mainly produced by oxidation of linoleic acid [49]. However, the relative content of linoleic acid increased significantly during the frying of squid, demonstrating a low level of oxidation. Moreover, the *trans*, *trans*-2,4-heptadienal, which was hardly detected after frying treatment at 160 °C increased after frying treatment at 180 °C, which initially demonstrated the temperature dependence of the concentration change.

As can be seen from Table 3 and Table 4, most of the aldehyde concentrations in squid lipids followed a time-dependent pattern. An increase in aldehyde concentration with extended frying time could be observed at both 160 °C and 180 °C, and this trend was particularly significant at 10 min. This was consistent with previous findings, which indicated that aldehyde generation from chicken breast, fish, and pork lipid oxidation intensified with extended thermal processing temperature and time [35,50,51]. However, some aldehydes, such as propanal, nonanal, and *trans*, *trans*-2,4-decadienal (DDE), showed a decrease in concentration with increasing time. Specifically, the concentrations of propanal were 23.17 ± 0.67, 22.32 ± 0.26, and 21.11 ± 0.43 μg/g at 160 °C frying treatment for 6 min, 8 min, and 10 min, respectively. The concentrations of nonanal were 22.62 ± 2.71 and 18.34 ± 4.09 μg/g at 160 °C frying treatment for 6 min, 8 min, respectively, and 8.15 ± 2.12 and 7.93 ± 2.14 μg/g at 180 °C frying treatment for 6 min and 8 min, respectively. The concentrations of DDE were 0.81 ± 0.07 and 0.62 ± 0.13 μg/g at 180 °C frying treatment for 8 min and 10 min, respectively. Previous studies have found similar results. Xu et al. [52] found that the concentrations of hexanal, nonanal, and *trans*, *trans*-2,4-heptadienal in linseed oil during French fries decreased with time. Zhang et al. [35] found that the concentration of volatile aldehydes during soybean oil frying increased and then decreased with time. These results indicated that excessive frying time could lead to the loss of aldehydes, especially volatile aldehydes, resulting in an aldehyde content decrease.

**Figure 3:**
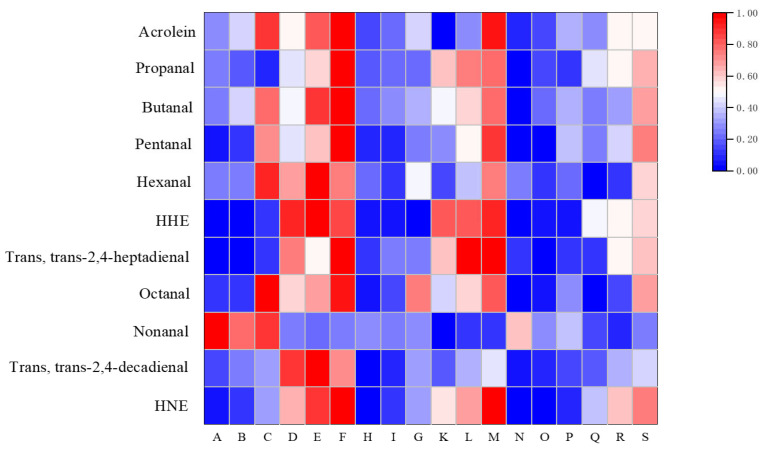
Heat map of changes in aldehyde content in squid lipids. A–F represent squid samples fried at 160 °C and 180 °C for 6 min, 8 min, and 10 min, respectively. H–M represent the squid samples fried under the same conditions after treatment with EDTA-2Na. N–S represent the squid samples fried under the same conditions after treatment with CEF.

As can be seen in Figure 3, the changes in aldehyde concentration in squid lipids during frying also indicated temperature dependence, and the aldehyde concentration increased significantly with the increase in temperature. Among them, the concentrations of pentanal, HHE, DDE, and HNE increased especially significantly with temperature. In particular, the concentration of pentanal was 1.31, 1.54, and 0.32 folds higher in the 180 °C frying treatment than in the 160 °C after the same time, respectively. The concentration of HHE was 1.91, 1.92, and 1.26 folds higher in the 180 °C frying treatment than in the 160 °C frying treatment under the same time condition, respectively. The concentration of DDE was 2.48, 2.00, and 0.91 folds higher in the 180 °C frying treatment than in the 160 °C frying treatment under the same time condition, respectively. The concentration of HNE of the 180 °C frying sample was 1.09, 1.27, and 0.90 folds higher than the 160 °C frying group, respectively, after the same time condition. A number of studies have confirmed the reliability of this result. Zhuang et al. [53] found that the concentrations of MDA, HHE, and HNE in palm oil during heating increased significantly with temperature. Luciane et al. [54] also found that the concentration of α, β-unsaturated aldehydes in soybean oil was higher at high temperatures than at low temperatures. Interestingly, some aldehydes showed the opposite trend. For example, the concentration of hexanal at 10 min of frying at 180 °C was lower than that at 10 min of frying at 160 °C. The concentrations of nonanal at 6 min, 8 min, and 10 min of frying at 180 °C were significantly lower than those at the same time of frying at 160 °C. Similarly, Liu et al. [17] reported that the concentrations of aldehydes, like 2-hexenal, decreased under high-temperature frying conditions of clams. Meinert et al. [55] also found that MDA content in pork lipids decreased under higher frying temperature. These results suggested that high temperature readily leads to significant loss of some aldehydes, resulting in the decrease of aldehyde. Logically, this included volatilization of aldehydes at elevated temperatures; reactions of aldehydes with proteins, amino acids, and nucleic acids; depletion of fatty acid substrates; and exchange of aldehydes between the squid and the frying oil.

Consistent with the results of fatty acid composition alteration in squid, both CEF and EDTA-2Na treatment inhibited the production and accumulation of aldehydes in squid, and the inhibitory effect of CEF was significantly better than that of EDTA-2Na. For example, EDTA-2Na-treated squid inhibited HHE by 9.05% and 12.71% when fried at 180 °C for 6 min and 8 min, respectively, with a non-significant inhibitory effect at 10 min, and HNE by 8.44% and 15.23% at 10 min, respectively, with a non-significant inhibitory effect at 10 min. CEF treatment inhibited HHE by 31.90%, 33.24%, and 19.73%, and HNE by 22.65%, 18.96%, and 17.28% when fried at 180 °C for 6 min, 8 min, and 10 min, respectively. Reports in the literature have identified the role of natural polyphenols in inhibiting the generation and accumulation of aldehydes. The mechanisms mainly include scavenging free radicals due to the role of electron donor, blocking chain reactions, chelating metal ions, and trapping aldehydes to produce adducts [56]. Hu et al. [27] observed that polyphenolic compounds in bamboo leaves significantly inhibited the production of various aldehydes during scallop frying. Banerjee et al. [57] found that marinating potatoes with curcumin significantly reduced HNE production during frying. In summary, CEF, as a member of natural polyphenolic compounds, proved its excellent antioxidant capacity by inhibiting the production and accumulation of aldehydes in fried squid.

## 4. Conclusions

Pan-frying processing of squid caused severe oxidation of squid lipids and contributed to a significant increase in PV, TBARS, and TOTOX values. PUFAs, especially EPA and DHA, underwent serious oxidative deterioration and resulted in massive aldehyde generation in a time- and temperature-dependent pattern, especially HHE and HNE, which are harmful to the human body. CEF and EDTA-2Na treatment were both able to inhibit the oxidation of PUFAs and retard the reactive aldehyde generation. In comparison, CEF was more effective than the EDTA-2Na. Therefore, short time and low temperature processing conditions for the aquatic products should be taken into consideration, and the utilization of flavonoids provides an effective measure to inhibit the loss of nutritional value and improve the consumption security of thermally processed squid products.

## Figures and Tables

**Table 1:** Changes in fatty acid composition (%) of squid lipids during frying at 160 °C.

Fatty Acid	Fresh	Control-6	Control-10	EDTA-2Na-6	EDTA-2Na-10	CEF-6	CEF-10
C12:0	0.03 ± 0.01 ^a^	0.01 ± 0.00 ^bc^	0.01 ± 0.00 ^c^	0.01 ± 0.00 ^bc^	0.01 ± 0.00 ^c^	0.01 ± 0.00 ^b^	0.01 ± 0.00 ^bc^
C13:0	0.02 ± 0.00	nd	nd	nd	nd	nd	nd
C14:0	2.32 ± 0.56 ^a^	0.77 ± 0.02 ^c^	0.60 ± 0.01 ^d^	0.69 ± 0.07 ^cd^	0.64 ± 0.0 ^d^	0.92 ± 0.05 ^b^	0.71 ± 0.02 ^cd^
C14:1 n-5	0.03 ± 0.01 ^a^	0.01 ± 0.00 ^b^	0.01 ± 0.00 ^b^	0.01 ± 0.00 ^b^	0.01 ± 0.00 ^b^	0.01 ± 0.00 ^b^	0.01 ± 0.00 ^b^
C15:0	0.75 ± 0.02 ^a^	0.22 ± 0.01 ^b^	0.12 ± 0.00 ^d^	0.15 ± 0.02 ^bcd^	0.17 ± 0.01 ^bcd^	0.20 ± 0.00 ^bc^	0.14 ± 0.00 ^cd^
C16:0	33.28 ± 1.61 ^a^	16.64 ± 0.70 ^bc^	15.18 ± 0.10 ^c^	16.04 ± 1.34 ^c^	16.06 ± 0.52 ^c^	18.14 ± 0.25 ^b^	16.13 ± 0.77 ^c^
C16:1 n-7	0.58 ± 0.03 ^a^	0.23 ± 0.01 ^c^	0.23 ± 0.00 ^c^	0.22 ± 0.03 ^c^	0.19 ± 0.00 ^c^	0.30 ± 0.01 ^b^	0.23 ± 0.01 ^c^
C17:0	1.46 ± 0.02 ^a^	0.40 ± 0.01 ^b^	0.29 ± 0.01 ^b^	0.37 ± 0.05 ^b^	0.36 ± 0.01 ^b^	0.47 ± 0.01 ^b^	0.35 ± 0.02 ^b^
C18:0	10.63 ± 0.60 ^a^	3.69 ± 0.16 ^b^	3.41 ± 0.05 ^b^	3.93 ± 0.42 ^b^	3.64 ± 0.14 ^b^	4.45 ± 0.16 ^b^	3.79 ± 0.21 ^b^
C18:1 n-9	2.60 ± 0.25 ^d^	20.02 ± 1.31 ^b^	22.10 ± 0.38 ^a^	20.37 ± 1.98 ^b^	20.99 ± 0.72 ^ab^	17.69 ± 0.90 ^c^	20.51 ± 1.18 ^b^
C18:2 n-6	0.25 ± 0.03 ^c^	45.14 ± 2.88 ^a^	48.30 ± 0.99 ^a^	45.56 ± 3.78 ^a^	47.02 ± 1.55 ^a^	41.26 ± 2.03 ^b^	46.74 ± 2.54 ^a^
C18:3 n-3	0.13 ± 0.02 ^d^	0.62 ± 0.03 ^b^	0.73 ± 0.02 ^a^	0.65 ± 0.08 ^b^	0.68 ± 0.02 ^ab^	0.50 ± 0.03 ^c^	0.65 ± 0.04 ^b^
C20:0	0.16 ± 0.02 ^f^	0.33 ± 0.03 ^c^	0.40 ± 0.00 ^a^	0.36 ± 0.04 ^bc^	0.37 ± 0.01 ^ab^	0.29 ± 0.02 ^d^	0.35 ± 0.02 ^bc^
C20:1 n-9	1.59 ± 0.19 ^a^	0.54 ± 0.02 ^b^	0.52 ± 0.02 ^b^	0.62 ± 0.07 ^b^	0.56 ± 0.02 ^b^	0.66 ± 0.01 ^b^	0.54 ± 0.03 ^b^
C20:2 n-6	0.28 ± 0.01 ^a^	0.09 ± 0.00 ^b^	0.06 ± 0.00 ^b^	0.07 ± 0.01 ^b^	0.08 ± 0.00 ^b^	0.09 ± 0.00 ^b^	0.07 ± 0.00 ^b^
C20:3 n-3	0.12 ± 0.02 ^a^	0.03 ± 0.00 ^b^	0.02 ± 0.00 ^b^	0.02 ± 0.00 ^b^	0.02 ± 0.00 ^b^	0.03 ± 0.00 ^b^	0.03 ± 0.00 ^b^
C20:4 n-6	7.70 ± 0.54 ^a^	1.72 ± 0.05 ^b^	1.23 ± 0.05 ^b^	1.77 ± 0.19 ^b^	1.51 ± 0.05 ^b^	2.58 ± 0.01 ^b^	1.46 ± 0.08 ^b^
C20:5 n-3	8.93 ± 0.01 ^a^	2.17 ± 0.08 ^bc^	1.64 ± 0.03 ^c^	2.19 ± 0.24 ^bc^	1.72 ± 0.06 ^c^	3.02 ± 0.05 ^b^	2.01 ± 0.12 ^bc^
C22:0	0.65 ± 0.02 ^a^	0.23 ± 0.01 ^bc^	0.21 ± 0.00 ^c^	0.24 ± 0.03 ^bc^	0.21 ± 0.01 ^c^	0.28 ± 0.00 ^b^	0.22 ± 0.01 ^bc^
C22:1 n-9	nd	0.03 ± 0.00 ^b^	0.03 ± 0.00 ^b^	0.03 ± 0.00 ^b^	0.03 ± 0.00 ^b^	0.04 ± 0.00 ^a^	0.03 ± 0.00 ^b^
C22:6 n-3	28.37 ± 1.28 ^a^	7.02 ± 0.24 ^c^	4.80 ± 0.15 ^d^	6.60 ± 0.76 ^cd^	5.62 ± 0.20 ^cd^	8.99 ± 0.11 ^b^	5.93 ± 0.43 ^cd^
C24:0	0.12 ± 0.02 ^a^	0.09 ± 0.00 ^cd^	0.10 ± 0.00 ^bc^	0.10 ± 0.00 ^bc^	0.11 ± 0.00 ^ab^	0.08 ± 0.00 ^d^	0.09 ± 0.01 ^cd^
ΣPUFA	45.78 ± 0.57 ^b^	56.79 ± 0.13 ^a^	56.78 ± 0.06 ^a^	56.86 ± 0.32 ^a^	56.65 ± 0.09 ^a^	56.47 ± 0.04 ^a^	56.87 ± 0.11 ^a^
EPA + DHA	37.30 ± 0.41 ^a^	9.19 ± 0.16 ^c^	6.44 ± 0.10 ^f^	8.79 ± 0.58 ^c^	7.34 ± 0.16 ^e^	12.01 ± 0.10 ^b^	7.94 ± 0.30 ^d^

The data in the table are expressed in the form of “Mean ± SD” (n = 3), and different letters after each row of data indicate statistically significant differences (*p* < 0.05) of this fatty acid in different squid lipid samples; “nd” indicates not detected.

**Table 2:** Changes in fatty acid composition (%) of squid lipids during frying at 180 °C.

Fatty Acid	Fresh	Control-6	Control-10	EDTA-2Na-6	EDTA-2Na-10	CEF-6	CEF-10
C12:0	0.03 ± 0.01 ^a^	0.01 ± 0.00 ^c^	0.01 ± 0.00 ^c^	0.01 ± 0.00 ^bc^	0.01 ± 0.00 ^bc^	0.01 ± 0.00 ^b^	0.01 ± 0.00 ^bc^
C13:0	0.02 ± 0.00	nd	nd	nd	nd	nd	nd
C14:0	2.32 ± 0.56 ^a^	0.51 ± 0.01 ^c^	0.55 ± 0.01 ^c^	0.74 ± 0.00 ^bc^	0.80 ± 0.01 ^bc^	1.02 ± 0.00 ^b^	0.78 ± 0.03 ^bc^
C14:1 n-5	0.03 ± 0.01 ^a^	0.01 ± 0.00 ^c^	0.01 ± 0.00 ^c^	0.01 ± 0.00 ^bc^	0.01 ± 0.00 ^bc^	0.02 ± 0.00 ^b^	0.01 ± 0.00 ^bc^
C15:0	0.75 ± 0.02 ^a^	0.10 ± 0.00 ^f^	0.15 ± 0.00 ^d^	0.13 ± 0.00 ^e^	0.19 ± 0.00 ^b^	0.17 ± 0.00 ^c^	0.17 ± 0.01 ^cd^
C16:0	33.28 ± 1.61 ^a^	14.57 ± 0.02 ^d^	15.28 ± 0.04 ^d^	16.42 ± 0.06 ^c^	16.51 ± 0.04 ^c^	17.79 ± 0.15 ^b^	16.66 ± 0.21 ^c^
C16:1 n-7	0.58 ± 0.03 ^a^	0.18 ± 0.01 ^e^	0.20 ± 0.00 ^e^	0.29 ± 0.01 ^c^	0.25 ± 0.00 ^d^	0.39 ± 0.01 ^b^	0.26 ± 0.01 ^d^
C17:0	1.46 ± 0.02 ^a^	0.27 ± 0.00 ^f^	0.35 ± 0.00 ^d^	0.32 ± 0.00 ^e^	0.40 ± 0.00 ^b^	0.37 ± 0.00 ^c^	0.40 ± 0.02 ^b^
C18:0	10.63 ± 0.60 ^a^	3.36 ± 0.04 ^d^	3.53 ± 0.04 ^cd^	3.82 ± 0.04 ^bc^	4.02 ± 0.03 ^b^	3.99 ± 0.03 ^b^	4.17 ± 0.13 ^b^
C18:1 n-9	2.60 ± 0.25 ^f^	22.77 ± 0.20 ^a^	21.51 ± 0.10 ^b^	20.01 ± 0.13 ^c^	19.92 ± 0.04 ^c^	18.62 ± 0.12 ^e^	19.56 ± 0.28 ^d^
C18:2 n-6	0.25 ± 0.03 ^f^	49.01 ± 0.37 ^a^	47.81 ± 0.13 ^b^	45.24 ± 0.27 ^c^	44.58 ± 0.25 ^d^	42.83 ± 0.20 ^e^	44.64 ± 0.70 ^d^
C18:3 n-3	0.13 ± 0.02 ^f^	0.77 ± 0.02 ^a^	0.71 ± 0.01 ^b^	0.63 ± 0.00 ^c^	0.64 ± 0.01 ^c^	0.55 ± 0.01 ^e^	0.59 ± 0.01 ^d^
C20:0	0.16 ± 0.02 ^f^	0.43 ± 0.01 ^a^	0.39 ± 0.01 ^b^	0.36 ± 0.01 ^c^	0.36 ± 0.01 ^c^	0.31 ± 0.00 ^e^	0.33 ± 0.01 ^d^
C20:1 n-9	1.59 ± 0.19 ^a^	0.52 ± 0.01 ^d^	0.54 ± 0.01 ^cd^	0.66 ± 0.01 ^b^	0.65 ± 0.01 ^bc^	0.65 ± 0.01 ^bc^	0.58 ± 0.02 ^bcd^
C20:2 n-6	0.28 ± 0.01 ^a^	0.06 ± 0.00 ^d^	0.08 ± 0.00 ^b^	0.07 ± 0.00 ^c^	0.09 ± 0.00 ^b^	0.08 ± 0.00 ^b^	0.08 ± 0.00 ^b^
C20:3 n-3	0.12 ± 0.02 ^a^	0.02 ± 0.00 ^d^	0.02 ± 0.00 ^cd^	0.03 ± 0.00 ^bcd^	0.02 ± 0.00 ^cd^	0.03 ± 0.00 ^b^	0.03 ± 0.00 ^bc^
C20:4 n-6	7.70 ± 0.54 ^a^	1.02 ± 0.02 ^e^	1.34 ± 0.02 ^de^	1.56 ± 0.02 ^cd^	1.79 ± 0.03 ^bc^	1.81 ± 0.02 ^bc^	2.04 ± 0.11 ^b^
C20:5 n-3	8.93 ± 0.01 ^a^	1.55 ± 0.03 ^g^	1.75 ± 0.04 ^f^	2.59 ± 0.06 ^c^	2.33 ± 0.04 ^d^	2.99 ± 0.02 ^b^	2.23 ± 0.11 ^e^
C22:0	0.65 ± 0.02 ^a^	0.22 ± 0.00 ^e^	0.22 ± 0.00 ^e^	0.26 ± 0.00 ^c^	0.25 ± 0.00 ^d^	0.28 ± 0.00 ^b^	0.24 ± 0.01 ^d^
C22:1 n-9	nd	0.03 ± 0.00 ^d^	0.03 ± 0.00 ^bc^	0.03 ± 0.00 ^c^	0.03 ± 0.00 ^c^	0.04 ± 0.00 ^a^	0.04 ± 0.00 ^ab^
C22:6 n-3	28.37 ± 1.28 ^a^	4.48 ± 0.06 ^e^	5.41 ± 0.10 ^d^	6.71 ± 0.20 ^c^	7.04 ± 0.18 ^c^	7.95 ± 0.12 ^b^	7.08 ± 0.30 ^c^
C24:0	0.12 ± 0.02 ^a^	0.11 ± 0.00 ^bc^	0.11 ± 0.00 ^bc^	0.10 ± 0.00 ^bc^	0.10 ± 0.00 ^b^	0.09 ± 0.01 ^d^	0.09 ± 0.00 ^cd^
ΣPUFA	45.78 ± 0.57 ^b^	56.91 ± 0.31 ^a^	57.12 ± 0.09 ^a^	56.83 ± 0.21 ^a^	56.49 ± 0.18 ^a^	56.24 ± 0.17 ^a^	56.66 ± 0.56 ^a^
EPA + DHA	37.30 ± 0.41 ^a^	6.03 ± 0.05 ^e^	7.16 ± 0.08 ^d^	9.30 ± 0.16 ^c^	9.37 ± 0.14 ^c^	10.94 ± 0.08 ^b^	9.31 ± 0.23 ^c^

The data in the table are expressed in the form of “Mean ± SD” (n = 3), and different letters after each row of data indicate statistically significant differences (*p* < 0.05) of this fatty acid in different squid lipid samples; “nd” indicates not detected.

**Table 3:** Changes in aldehyde concentrations (μg/g) in the squid lipids during frying at 160 °C.

Compounds	A	B	C	D	E	F	G	H	I
Acrolein	0.04 ± 0.01 ^cd^	0.06 ± 0.01 ^bc^	0.13 ± 0.01 ^a^	0.02 ± 0.00 ^de^	0.03 ± 0.02 ^de^	0.06 ± 0.01 ^b^	0.01 ± 0.00 ^e^	0.02 ± 0.01 ^de^	0.05 ± 0.02 ^bc^
Propanal	23.17 ± 0.67 ^a^	22.32 ± 0.26 ^abc^	21.11 ± 0.43 ^de^	22.21 ± 0.54 ^abcd^	22.68 ± 0.58 ^ab^	22.62 ± 0.51 ^ab^	19.96 ± 0.21 ^e^	21.82 ± 0.41 ^bcd^	21.25 ± 1.59 ^cd^
Butanal	12.09 ± 0.69 ^c^	14.62 ± 0.94 ^b^	19.53 ± 3.27 ^a^	11.67 ± 0.39 ^c^	12.70 ± 1.16 ^bc^	13.40 ± 0.74 ^bc^	8.77 ± 1.02 ^d^	11.67 ± 0.91 ^c^	13.63 ± 0.72 ^bc^
Pentanal	18.84 ± 4.39 ^c^	20.99 ± 3.77 ^c^	59.37 ± 3.98 ^a^	19.51 ± 6.17 ^c^	19.76 ± 6.39 ^c^	31.38 ± 5.31 ^b^	14.59 ± 0.89 ^c^	15.62 ± 1.03 ^c^	38.89 ± 6.96 ^b^
*Trans*-2-hexenal	nd	nd	nd	nd	nd	nd	nd	nd	nd
Hexanal	4.03 ± 0.19 ^c^	4.04 ± 0.05 ^c^	7.03 ± 0.76 ^a^	3.77 ± 0.57 ^c^	3.35 ± 0.27 ^c^	5.05 ± 0.44 ^b^	3.98 ± 0.45 ^c^	3.33 ± 1.02 ^c^	3.86 ± 0.49 ^c^
HHE	73.43 ± 2.29 ^b^	77.06 ± 3.10 ^b^	90.06 ± 3.92 ^a^	78.72 ± 9.64 ^b^	77.94 ± 1.84 ^b^	72.00 ± 5.04 ^b^	74.67 ± 2.71 ^b^	80.65 ± 7.43 ^ab^	77.41 ± 3.79 ^b^
*Trans*, *trans*-2,4-heptadienal	nd	nd	0.01 ± 0.00 ^a^	0.01 ± 0.01 ^a^	0.02 ± 0.01 ^a^	0.02 ± 0.01 ^a^	0.01 ± 0.01 ^a^	nd	0.01 ± 0.00 ^a^
Octanal	1.70 ± 0.16 ^bc^	1.72 ± 0.06 ^bc^	3.39 ± 0.53 ^a^	1.57 ± 0.36 ^bc^	1.76 ± 0.39 ^bc^	2.91 ± 0.30 ^a^	1.49 ± 0.30 ^c^	1.56 ± 0.49 ^bc^	2.05 ± 0.18 ^b^
Nonanal	22.62 ± 2.71 ^a^	18.34 ± 4.09 ^ab^	20.31 ± 5.4 ^a^	8.76 ± 0.63 ^c^	8.11 ± 1.26 ^c^	9.22 ± 1.33 ^c^	15.65 ± 1.79 ^b^	9.03 ± 0.73 ^c^	10.76 ± 1.91 ^c^
DDE	0.21 ± 0.07 ^abc^	0.27 ± 0.05 ^ab^	0.33 ± 0.20 ^a^	0.10 ± 0.68 ^c^	0.16 ± 0.59 ^bc^	0.33 ± 0.46 ^a^	0.13 ± 0.32 ^bc^	0.15 ± 0.12 ^bc^	0.21 ± 0.55 ^abc^
HNE	8.27 ± 1.08 ^bc^	9.22 ± 0.50 ^b^	12.06 ± 1.12 ^a^	7.60 ± 1.09 ^bc^	9.11 ± 0.82 ^bc^	11.97 ± 2.64 ^a^	7.23 ± 0.08 ^c^	7.33 ± 0.53 ^bc^	8.79 ± 0.15 ^bc^

The data in the table are expressed in the form of “Mean ± SD” (n = 3), and different letters after each row of data indicate statistically significant differences (*p* < 0.05) of this fatty acid in different squid lipid samples; “nd” indicates that it was not detected. A–C represent squid samples fried at 160 °C for 6 min, 8 min, and 10 min, respectively. D–F represent the squid samples fried under the same conditions after treatment with EDTA-2Na. G–I represent the squid samples fried under the same conditions after CEF treatment. ^a–e^ Values in the same line with different lower-case letters are significantly different at *p* < 0.05.

**Table 4:** Changes in aldehyde concentrations (μg/g) in the squid lipids during frying at 180 °C.

Compounds	A	B	C	D	E	F	G	H	I
Acrolein	0.08 ± 0.01 ^c^	0.12 ± 0.01 ^b^	0.15 ± 0.00 ^a^	nd	0.04 ± 0.01 ^d^	0.14 ± 0.01 ^ab^	0.04 ± 0.01 ^d^	0.08 ± 0.02 ^c^	0.08 ± 0.01 ^c^
Propanal	25.86 ± 0.42 ^cd^	27.49 ± 1.13 ^bcd^	32.68 ± 1.22 ^a^	27.97 ± 1.44 ^bcd^	29.70 ± 0.54 ^b^	30.09 ± 3.79 ^ab^	25.60 ± 0.36 ^d^	26.63 ± 1.47 ^cd^	28.39 ± 0.49 ^bc^
Butanal	15.39 ± 1.86 ^de^	20.71 ± 3.76 ^ab^	22.30 ± 1.10 ^a^	15.31 ± 0.47 ^de^	16.61 ± 0.95 ^cd^	19.45 ± 2.80 ^abc^	12.08 ± 0.58 ^f^	13.06 ± 0.78 ^ef^	17.99 ± 0.64 ^bcd^
Pentanal	43.44 ± 8.06 ^d^	53.58 ± 7.54 ^c^	78.36 ± 2.32 ^a^	32.18 ± 2.74 ^e^	46.57 ± 3.87 ^cd^	71.91 ± 7.00 ^ab^	30.08 ± 3.88 ^e^	41.51 ± 7.60 ^d^	63.46 ± 1.47 ^b^
*Trans*-2-hexenal	0.01 ± 0.00 ^b^	nd	nd	0.01 ± 0.00 ^a^	0.01 ± 0.00 ^b^	0.01 ± 0.00 ^b^	nd	nd	nd
Hexanal	5.91 ± 0.99 ^b^	7.41 ± 1.65 ^a^	6.27 ± 0.87 ^ab^	3.46 ± 0.20 ^de^	4.62 ± 0.44 ^cd^	6.33 ± 0.57 ^ab^	2.84 ± 0.64 ^e^	3.43 ± 0.40 ^de^	5.43 ± 0.22 ^bc^
HHE	214.41 ± 17.60 ^bc^	225.05 ± 13.09 ^a^	203.17 ± 28.49 ^bc^	195.01 ± 20.75 ^b^	196.45 ± 15.07 ^b^	211.00 ± 8.04 ^bc^	146.02 ± 22.40 ^c^	150.24 ± 7.51 ^c^	163.08 ± 7.57 ^c^
*Trans*, *trans*-2,4-heptadienal	0.06 ± 0.01 ^b^	0.04 ± 0.00 ^d^	0.08 ± 0.00 ^a^	0.05 ± 0.01 ^bcd^	0.08 ± 0.02 ^a^	0.08 ± 0.01 ^a^	0.01 ± 0.01 ^e^	0.04 ± 0.01 ^cd^	0.05 ± 0.00 ^bc^
Octanal	2.61 ± 0.16 ^cd^	2.81 ± 0.23 ^bc^	3.27 ± 0.16 ^a^	2.29 ± 0.17 ^d^	2.62 ± 0.23 ^cd^	3.03 ± 0.18 ^ab^	1.55 ± 0.40 ^e^	1.76 ± 0.32 ^e^	2.82 ± 0.19 ^bc^
Nonanal	8.15 ± 2.12 ^a^	7.93 ± 2.14 ^ab^	8.23 ± 1.40 ^a^	3.69 ± 0.52 ^d^	5.77 ± 0.77 ^bcd^	5.77 ± 1.27 ^bcd^	6.49 ± 0.83 ^abc^	5.44 ± 0.94 ^cd^	8.22 ± 0.81 ^a^
DDE	0.73 ± 0.26 ^a^	0.81 ± 0.07 ^a^	0.62 ± 0.13 ^ab^	0.23 ± 0.13 ^c^	0.36 ± 0.11 ^c^	0.42 ± 0.11 ^bc^	0.22 ± 0.32 ^c^	0.36 ± 0.12 ^c^	0.40 ± 0.05 ^c^
HNE	17.30 ± 2.66 ^d^	20.94 ± 2.46 ^abc^	22.92 ± 2.17 ^a^	15.84 ± 1.55 ^de^	17.75 ± 1.04 ^cd^	22.54 ± 0.57 ^ab^	13.38 ± 0.72 ^e^	16.97 ± 2.92 ^de^	18.96 ± 3.17 ^bcd^

The data in the table are expressed in the form of “Mean ± SD” (n = 3), and different letters after each row of data indicate statistically significant differences (*p* < 0.05) of this fatty acid in different squid lipid samples; “nd” indicates that it was not detected. A–C represent squid samples fried at 180 °C for 6 min, 8 min, and 10 min, respectively. D–F represent the squid samples fried under the same conditions after treatment with EDTA-2Na. G–I represent the squid samples fried under the same conditions after treatment with CEF. ^a–f^ Values in the same line with different lower-case letters are significantly different at *p* < 0.05.

## Data Availability

The original contributions presented in this study are included in the article. Further information can be available after inquiries to the corresponding author.

## Abbreviations

PUFA	polyunsaturated fatty acids
EDTA-2Na	disodium ethylenediaminetetraacetate
n-3 PUFA	omega-3 PUFA
EPA	eicosapentaenoic acid
DHA	docosahexaenoic acid
HNE	4-hydroxy-nonenal
CEF	flavonoids extracted from coconut exocarp
DNPH	2,4-dinitrophenylhydrazine
PV	peroxide value
TBARS	thiobarbituric acid-reactive substances
MDA	malondialdehyde
TOTOX	total oxidation
GC-MS	gas chromatography–mass spectrometry
HPLC	high-performance liquid chromatography
SD	standard deviation
HHE	pentanal, 4-hydroxy-hexenal
DDE	trans, trans-2,4-decadienal
